# Ecological disequilibrium drives insect pest and pathogen accumulation in non-native trees

**DOI:** 10.1093/aobpla/plw081

**Published:** 2016-12-23

**Authors:** Casparus J. Crous, Treena I. Burgess, Johannes J. Le Roux, David M. Richardson, Bernard Slippers, Michael J. Wingfield

**Affiliations:** 1 Forestry and Agricultural Biotechnology Institute (FABI), University of Pretoria, Pretoria, 0002, South Africa; 2 Present address: Centre for Ecology, Evolution and Environmental Changes, Faculty of Sciences, University of Lisbon, Campo Grande, Lisbon, 1749-016, Portugal; 3 Centre for Phytophthora Science and Management, School of Veterinary and Life Sciences, Murdoch University, 90 South Street, Murdoch, WA, 6150, Australia; 4 Department of Botany and Zoology, Centre for Invasion Biology, Stellenbosch University, Private Bag X1, Matieland, 7602, South Africa

**Keywords:** Acacia, biological control, biological invasions, biosecurity, eco-evolutionary experience, eucalyptus, herbivore host-shifting, phylogenetic relatedness, Pinus

## Abstract

Non-native trees have become dominant components of many landscapes, including urban ecosystems, commercial forestry plantations, fruit orchards and as invasives in natural ecosystems. Often, these trees have been separated from their natural enemies (i.e. insects and pathogens) leading to ecological disequilibrium, that is, the immediate breakdown of historically co-evolved interactions once introduced into novel environments. Long-established, non-native tree plantations provide useful experiments to explore the dimensions of such ecological disequilibria. We quantify the *status quo* of non-native insect pests and pathogens catching up with their tree hosts (planted *Acacia*, *Eucalyptus* and *Pinus* species) in South Africa, and examine which native South African enemy species utilize these trees as hosts. Interestingly, pines, with no confamilial relatives in South Africa and the longest residence time (almost two centuries), have acquired only one highly polyphagous native pathogen. This is in contrast to acacias and eucalypts, both with many native and confamilial relatives in South Africa that have acquired more native pathogens. These patterns support the known role of phylogenetic relatedness of non-native and native floras in influencing the likelihood of pathogen shifts between them. This relationship, however, does not seem to hold for native insects. Native insects appear far more likely to expand their feeding habits onto non-native tree hosts than are native pathogens, although they are generally less damaging. The ecological disequilibrium conditions of non-native trees are deeply rooted in the eco-evolutionary experience of the host plant, co-evolved natural enemies and native organisms from the introduced range. We should expect considerable spatial and temporal variation in ecological disequilibrium conditions among non-native taxa, which can be significantly influenced by biosecurity and management practices.

## Teaser

Non-native plant invasions represent ecological disequilibrium situations, that is, the immediate breakdown of historically co-evolved interactions once introduced into novel environments. Globalization has led to increased transfer in organisms between countries. Native enemies also undergo host expansions onto non-native plants. Ecological disequilibrium conditions can thus be expected to change unpredictably over time. By retrospectively analysing the insect pest and pathogen accumulation on established non-native trees in South Africa, we review the eco-evolutionary background that may help to explain and predict variation in ecological disequilibrium conditions in non-native trees.

## Introduction

Introduced organisms can be seen as representing ecological disequilibrium situations, in that abiotic and biotic interactions and adaptations nurtured over evolutionary time scales in their native ranges are disrupted upon movement to novel introduced ranges. Such ecological disequilibrium conditions can enhance the invasiveness of some species, e.g. through release from specialist, co-evolved enemies and competitors found in their native habitat (e.g. Enemy Release Hypothesis, Keane and Crawley 2002). Absences of natural enemies could lead to re-investment of costly defence mechanisms into reproductive effort (i.e. Evolution of Increased Competitive Ability Hypothesis, Blossey and Nötzold 1995). Disequilibrium conditions are expected to change over time, as components of biotic interaction networks are reunited through on-going introductions and/or by novel interactions in the introduced range (Vacher *et al.*, 2010). This rate of change over time, in turn, is expected to be impacted by the relatedness of introduced taxa to the recipient community’s biota. For example, invaded communities harbouring species phylogenetically closely related to the introduced species could act as reservoirs for pre-adapted enemies (Parker and Gilbert 2004; Ness *et al.*, 2011).

The concept of eco-evolutionary experience (EEE) can help to explain invasion success (Saul *et al.*, 2013; Saul and Jeschke 2015). This concept predicts enhanced probability of an organism becoming invasive because enemy species in the novel environment have little EEE to either perceive the invader as a potential resource, or they are unable to utilize it (e.g. the introduced species might have unique herbivore defence strategies). Furthermore, although not explicitly defined as part of the original EEE concept, conceivably the similarity of the environmental conditions in which the invading organism evolved and resided may strengthen the likelihood of establishment and invasion, i.e. being pre-adapted (Facon *et al.*, 2006). Against this background, variation in ecological disequilibrium conditions among non-native organisms appears intrinsic to the biotic and abiotic EEE of the non-native organism in the introduced range.

Intentional, large-scale introductions of diverse species variably related to the native flora, represent a natural experiment to investigate enemy release and accumulation over time (Flory and Clay 2013; Flory and D’Antonio 2015; Burgess and Wingfield 2017), and how phylogenetic relatedness impacts on these. Such a scenario exists in South Africa where various unrelated non-native trees in the genera *Acacia*, *Eucalyptus* and *Pinus* were introduced, starting in earnest in the 1800s, to supply a growing demand for wood, wood related products, and for ecological restoration (King 1943; Burgess and Wingfield 2001; Richardson *et al.*, 2003). Introduced species in the genera *Pinus* (Gymnospermae), *Eucalyptus* and *Acacia* (Angiospermae) vary in level of relatedness with the South Africa biota. If we assume that a higher degree of phylogenetic relatedness to native flora is a proxy for eco-evolutionary similarity, then the interest of the experiment is increased as it allows us to explore the extent to which the likelihood of novel species interactions becoming established is related to the evolutionary relatedness of both donor and recipient communities (Parker and Gilbert 2004; Mitchell *et al.*, 2006). Therein, focusing on associated insect and pathogen pests of invasive species is of particular interest for inferences about enemy release, since these organisms cause severe damage to plant populations (Mitchell and Power 2003).

Increasingly, non-native plantation trees are being affected by non-native herbivores, i.e. herbivores originating from outside the introduced range, including the host plant’s native range (Wingfield *et al.*, 2008, 2015; Hurley *et al.*, 2016). For example, co-evolved insect pests of Australian eucalypts are increasingly observed in plantations globally (Paine *et al.*, 2011; Hurley *et al.*, 2016). Imported Australian acacias and eucalypts in South Africa have experienced a gradual increase in non-native pathogens ‘catching up’ with their ‘lost’ hosts (Wingfield *et al.*, 2011). Thus, historical (co-evolved) biotic interactions are accumulating in space and time (Wingfield *et al.*, 2011; Flory and Clay 2013). Accordingly, the enemy-free space, characterizing invasion into novel environments, could be considered as shrinking over time (Jeffries and Lawton 1984).

Native herbivores and pathogens can also undergo host expansions onto non-native plants (Jaenike 1983, 1990; Parker and Hay 2005; Bezemer *et al.*, 2014; Cahenzli *et al.*, 2015). For example, the South African native legume, *Virgilia divaricata*, shares up to a third of its total arthropod community with the confamilial introduced and invasive *Acacia mearnsii* (van der Colff *et al.*, 2015). Procheş *et al.* (2008) found high abundances of native herbivores on a wide range of non-native trees in fynbos flora. *Chrysoporthe austroafricana*, a fungus native on the indigenous tree *Syzygium cordatum* (Heath *et al.*, 2006), has caused a serious stem canker disease on con-familial non-native *Eucalyptus* species in South Africa (Wingfield *et al.*, 1989).

In summary, the presence of native and non-native insect pests and pathogens on acacias, eucalypts and pines has been well studied in South Africa (Wingfield *et al.*, 2008; Roux *et al.*, 2012). Species in these three genera were introduced without their natural enemies. In this paper, we review the *status quo* of non-native insect pests and pathogens catching up with their associated hosts, and also of those native insect pests and pathogens starting to utilize these introduced trees as a resource (host shifts). We further considered whether native and introduced pest and pathogen communities are polyphagous or more host-specific across acacias, eucalypts and pines. The overall aim was to conceptualize the ecological and evolutionary background that may help to explain and predict changes in biotic interactions underlying invasive tree populations.

## Historic and Novel Biotic Interactions on Non-Native *Eucalyptus*, *Acacia* and *Pinus* Species

### General overview

**Figure 1.**
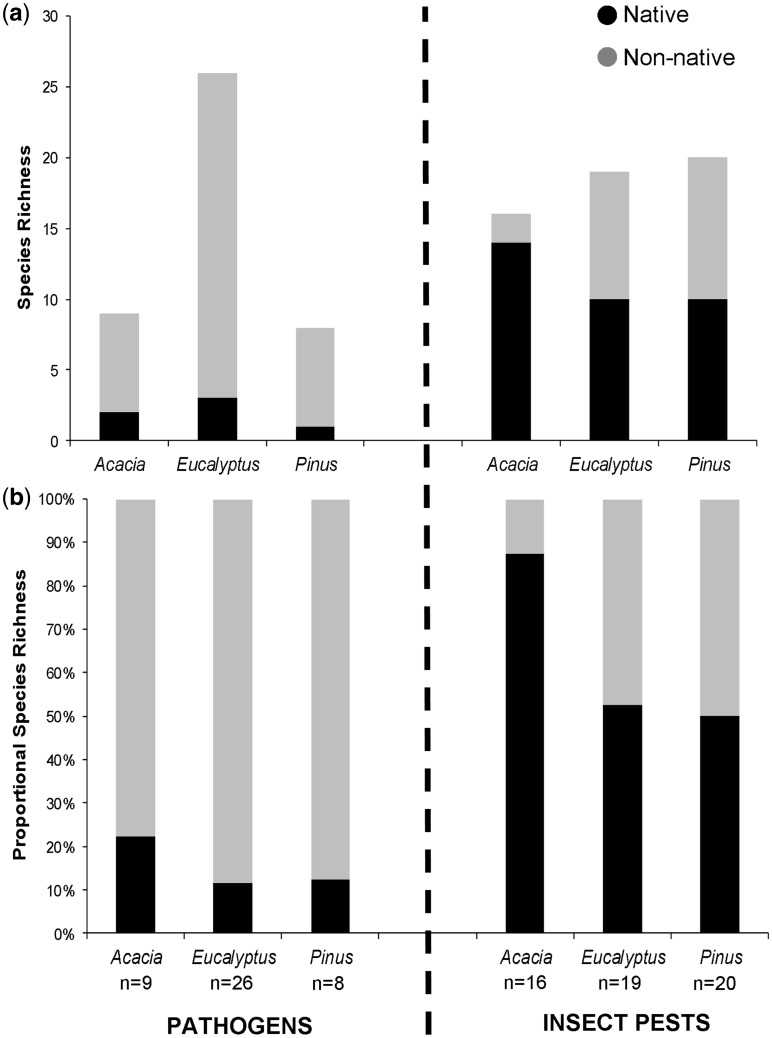
(A) Native and non-native insect pest and pathogen accumulation onto *Acacia*, *Eucalyptus* and *Pinus* plantations in South Africa (data from Wingfield *et al.* 2008 and Roux *et al.* 2012). (B) For insect pest and pathogen categories, the proportion of native species compared to the total is also shown. For pathogens, the proportion of non-native catch-up events was markedly higher than for every native host shift event. For insect pests, catch-up and host-shift events accumulated at a more equal proportion, except for acacias, which appeared to have only few non-native insect pests. Large scale acacia plantations began in 1864, eucalypt plantations in 1828, and pine plantations in 1825.

Pine plantations cover roughly 51 % of all plantation areas in South Africa, eucalypts about 41 %, and acacias about 8  % (State of the Forests Report 2010–2012, Republic of South Africa, available online at http://www.nda.agric.za (13 April 2016)). Using data from inventories carried out in plantations over many decades (Wingfield *et al.*, 2008; Roux *et al.*, 2012), it appears that eucalypts had the highest number of native and non-native insect pests and pathogens affecting their health (*n* = 45; Fig. 1A), followed by pines (*n* = 28) and acacias (*n* = 25). The differences in insect pest and pathogen patterns among these three genera are unrelated to the total surface area of plantations in South Africa (Observed vs. Expected *X*^2 ^=^ ^48.71, *df* = 2, *P* <0.001; Table 1). However, when treating insect pests and pathogens separately, the observed number of catch-up events by non-native insect pests did track the plantation area (Observed vs. Expected *X*^2 ^=^ ^0.40, *df* = 2, *P* = 0.819; Table 1). The extent of plantations thus seems to be an inaccurate proxy for predicting the level of pathogen and native insect accumulation.

**Table 1.** Enemy accumulation on *Acacia*, *Eucalyptus* and *Pinus* species in South Africa in relation to area planted of each genus.*

Group	*X*^2^	*df*	*P*-value
**Overall enemy accumulation**	48.71	2	<0.001
***Pests***			
** Non-native**	0.40	2	0.819
** Native**	148.41	2	<0.001
**Pathogens**			
** Non-native**	46.02	2	<0.001
** Native**	105.09	2	<0.001

* Observed frequencies for goodness-of-fit tests were based on Aca*cia* covering 8 %, *Eucalyptus* 41 % and *Pinus* 51 % of the planted area. Calculations were carried out in Statistica 12 (Statsoft, Inc.).

The residence time of non-native tree species in the country might also be considered as a factor for predicting the number of catch-up events. In South Africa, planting of pines began in 1825, eucalypts around 1828, whereas widespread plantings of acacias, in particular *A. mearnsii*, began around 1864 (King 1943; Burgess and Wingfield 2001; see also Sappi Tree Farming Guidelines, Part 2, Silviculture, available at  https://cdn-s3.sappi.com/s3fs-public/Part-2-Silviculture.pdf (13 April 2016)). Acacias used in forestry could, therefore, be considered the most recent introductions. However, the accumulation of insect pest and pathogen catch-up events as observed across these three genera does not appear to track residence time *per se*, i.e. time since large-scale planting began (Fig. 1A). Instead, cumulative insect pest and pathogen richness was more likely dependent on the presence of a particular planted genus (Test of Independence *X*^2 ^=^ ^6.83, *df* = 2, *P* = 0.033; data not shown).

### Patterns of catch-up and host-shifts by pathogens

The number of non-native pathogens catching up with their hosts in South African populations differed between the studied tree genera. Considerably greater numbers of non-native pathogen catch-ups were recorded on eucalypts (*n* = 23) than on acacias and pines (both *n* = 7). For native South African pathogens, it was remarkable to see the low proportion recorded across all three genera (Fig. 1B). In particular, the approximately 300 years since the introduction of pines in South Africa (and 200 years of widespread commercial plantations), the only pathogen infecting pines and known to be native is *Armillaria fuscipes* (Coetzee *et al.*, 2000, 2005). *Armillaria* spp. are root rot pathogens that are recognized to have very wide host ranges (Raabe 1962; Hood *et al.*, 1991). In fact, *A. fuscipes* is known to infect all three of the genera under review (Roux *et al.*, 2012).

Whether pathogens have broad or narrow host ranges is of importance, as those with broad host range could be predicted, in the future, to infect a wide range of both native and non-native plants. Pooled native and non-native pathogen data indicated pines had the lowest number of shared pathogens compared to eucalypts and acacias (Table 2), with the latter two groups sharing a quarter of the total pathogen community. Thus, the pathogen dynamics for pines are unique in South Africa compared to the other two genera; not only are native pathogens relatively less likely to infect pines, but these conifers are also unaffected from a subset of non-native pathogens shared by acacias and eucalypts (Table 2).

**Table 2.** Community similarity (in percentage) of native and non-native (pooled) insect pests and pathogens shared between *Acacia*, *Eucalyptus* and *Pinus* species in South Africa.*

Genera	Microbial pathogens	Insect pests
***Pinus* vs. *Eucalyptus***	3 %	22 %
***Pinus* vs. *Acacia***	6 %	21 %
***Eucalyptus* vs. *Acacia***	25 %	17 %

* Percentage community similarity was calculated in PRIMER 6 (PRIMER-E, Lutton, UK) using a presence/absence matrix and Jaccard similarity distances.

### Patterns of catch-up and host-shift by insect pests

The low numbers of native pathogen host-shifts stands in stark contrast to the high incidences of native South African insects now associated with acacias, eucalypts and pines (Fig. 1A). Further, native insect host-shifts and non-native catch-ups appear to have accrued at similar levels. The only exception was for acacias, which had the highest number of associated native South African insect herbivores (*n* = 14, compared to 10 each for pines and eucalypts), but only two non-native insect pests catching up. The latter are the auger beetle, *Sinoxylon bellicosum*, and the shot-hole borer, *Apate indistincta*. Both species are considered natives to the African continent but putative non-natives to South Africa (introductions of acacia pests from Australia appear to be non-existent in South African plantations), and are not specifically associated with the genus *Acacia* (*sensu lato*). This is true even though *A. indistincta* has been noted to feed on *Pericopsis elata*, another member of the Fabaceae from Ghana (Bourland *et al.*, 2012). Native pathogens thus appear to be more host-specific than their native insect counterparts.

Native and non-native insect pests associated with non-native acacias, eucalypts and pines had strikingly different species assemblage patterns. Many native insect pests were shared among the three genera, while they did not share any of the non-native insect pests (Table 3). Indeed, we are not aware of any evidence suggesting these non-native insect pests have subsequently moved to other tree species within plantation matrices (see also Moran *et al.*, 2005). As such, non-native insect pest accumulation appears highly genus-specific. In turn, many native polyphagous insect pests appear capable of utilizing a phylogenetically diverse assemblage of non-native hosts. Overall, almost a quarter of the listed native insect pests fed on all three genera. Interestingly, eucalypts and pines shared the highest number of native pests (50 %), with acacias and pines ranking second in pest community similarity (39 %), and the two more closely related angiosperm genera third (33 %; Table 3).

**Table 3.** The percentage of native and non-native insect pests that are shared between *Acacia*, *Eucalyptus* and *Pinus* species in South Africa.*

Genera	Native pests	Non-native pests
***Pinus* vs. *Eucalyptus***	50 %	0 %
***Pinus* vs. *Acacia***	39 %	0 %
***Eucalyptus* vs. *Acacia***	33 %	0 %

* Percentage community similarity was calculated in PRIMER 6 (PRIMER-E, Lutton, UK) using a presence/absence matrix and Jaccard similarity distances.

## Loss of Enemy-Free Space in Non-Native Trees

### Pathogen accumulation

After almost 200 years of widespread commercial planting in South Africa, pines, the longest planted of the three genera included here, have only been infected by one highly polyphagous native pathogen, *A. fuscipes*. In contrast, *Acacia* and *Eucalyptus* species, both with shorter residence times, have been infected by more native pathogens. These latter native pathogens also appear to be more host-specific. Thus, the accumulation of native and relatively more host-specific pathogens might be constrained by factors other than the residence times of these three genera. One possible explanation for this pattern is the lack of native Pinaceae in southern Africa, and that the region is depauperate in extant conifers overall (Coates-Palgrave 2002). In contrast, southern Africa has 25 indigenous tree species in the Myrtaceae and about 80 indigenous tree species in the Fabaceae (Van Wyk and Van Wyk 1997).

Evidence for phylogenetic relatedness as a determining factor in host shifts of native pathogens onto non-native tree crops exists for many non-native trees in South Africa. For example, the native fungus *C. austroafricana* is an important stem canker pathogen in eucalypt plantations (Wingfield *et al.*, 1989; Nakabonge *et al.*, 2006). *Chrysoporthe austroafricana* occurs on the native tree genus *Syzygium*, which is in the same family as eucalypts (Heath *et al.*, 2006). Similarly, native Botryosphaeriaceae species found on *Syzygium* species have also been shown to infect *Eucalyptus* species (Pavlic *et al.*, 2007). Further, *Metrosideros angustifolia*, a fynbos endemic and also in the Myrtaceae, is often infected by a native pathogen, *Holocryphia capensis*, which can be pathogenic on *Eucalyptus grandis* (Chen *et al.*, 2016). Apart from these South African examples, pathogen host shifts between Myrtaceae are also observed elsewhere in the world. For example, in Uruguay, multiple native Botryosphariaceae fungi associated with Myrtaceae were isolated from non-native eucalypt plantations (Pérez *et al.*, 2010). Similarly, *Erwinia psidii*, a bacterial pathogen of the native South American tree *Psidium guajava*, has started to seriously infect eucalypt plantations in Uruguay and Argentina (Coutinho *et al.*, 2011).

For acacia plantations, the native fungus *Ceratocystis albifundus* causes a serious canker and wilt disease (Roux *et al.*, 2007; Wingfield *et al.*, 2011), while also killing native Fabaceae species such as *Senegalia caffra* (Roux *et al.*, 2007). The native fungus *Pseudolagarobasidium acaciicola* is suggested to be an opportunistic pathogen to various native Fabaceae (Kotzé *et al.*, 2015), and has subsequently been proposed as a possible mycoherbicide for invasive *Acacia cyclops* (Wood and Ginns 2006; Kotzé *et al.*, 2015).

**Figure 2.**
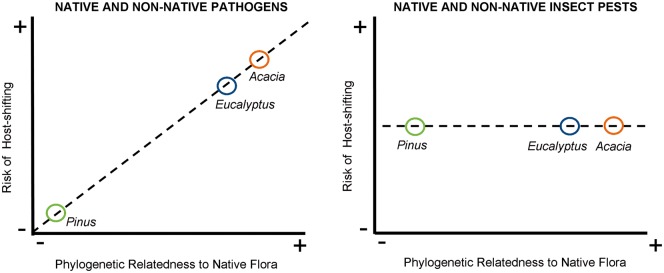
Conceptual diagram to help predict the risk of non-native insect pests and pathogens affecting native flora in South Africa via host-shifting events, as well as the likelihood that native insect pests and pathogens could affect non-native commercial tree crops. A key premise to such predictions is to take into account the phylogenetic relatedness of non-native tree species to the native flora (Parker and Gilbert 2004; Mitchell *et al.*, 2006). These predictions are based on plant health inventories of *Acacia*, *Eucalyptus* and *Pinus* plantations in South Africa. The risk of host-shifting by both native pathogens onto the non-native flora and non-native pathogens onto the native flora appears positively associated with the phylogenetic relationship of the introduced tree to the native flora. In contrast, there appears to be no association for both native and non-native insect pests. Being polyphagous drove this phylogenetically unrelated feeding pattern for native pests. However, being host-specific drove this phylogenetically unrelated feeding pattern in non-native insect pests (Table 3). This host-specificity pattern displayed by accidentally introduced insect pests is consistent with the target-specific herbivory habits of deliberately introduced agents for biological control (for example on *Acacia* species, Impson *et al.*, 2011).

Pines in South Africa have been devoid of pathogen attack by relatively host-specific organisms after many centuries of plantings. This observation is contrasted against non-native *P. radiata*, present in the habitat of the native *P. pinaster* in northwest Spain for less than 70 years, that has already accumulated two pine-specific native pathogens (Lombardero *et al.*, 2012). Thus, the phylogenetic relatedness of a non-native plant to the flora of the local community appears to be important for disentangling the variance in ecological disequilibrium in native pathogen accumulation between genera (see Fig. 2; Parker and Gilbert 2004).

Data collected for non-native pathogen catch-ups onto acacia, eucalypt and pine plantations in South Africa suggest very low pathogen-sharing at the host genus level (the highest pathogen-sharing was between acacias and eucalypts, at 25 %; Table 2). In line with this, across Europe, 77 % of 123 observed invasive forest pathogens were considered specialist and host-specific (Santini *et al.*, 2013). Thus, non-native pathogens invading into South African non-native plantations support the expected host-specificity pattern depicted by Santini *et al.* (2013) (Observed vs. Expected *X*^2 ^=^ ^0.23, *df* = 1, *P* = 0.635; data not shown). However, accidental introductions of polyphagous pathogens (e.g. *Phytophthora* species) are possible, increasing the probability of non-native pathogens infecting a wider variety of native and non-native flora.

**Figure 3.**
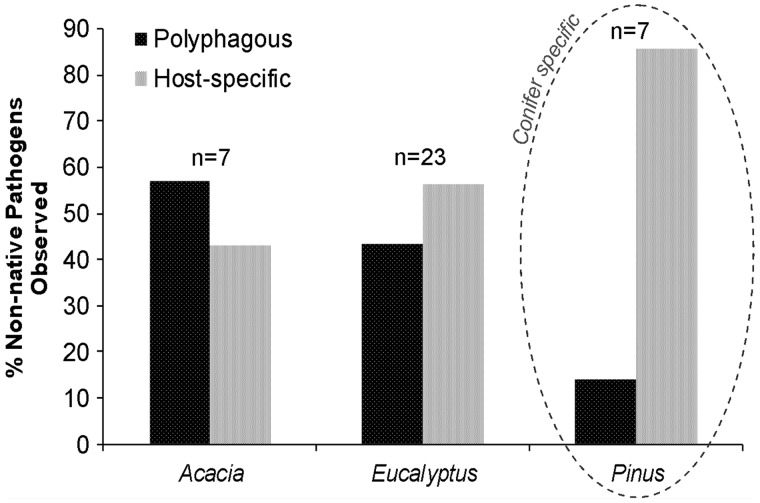
Known host-ranges of the non-native pathogens detected on *Acacia*, *Eucalyptus* and *Pinus* species in South African plantations. Host-specificity, in this instance, was defined as an organism that feeds within a plant family. Polyphagous refers to those organisms that feed across plant families. For *Pinus* species, none of the observed pathogens were known to be pathogenic on angiosperms. In turn, pathogens that fed on angiosperms may also feed on conifers [see Supporting Information—Table S1].

Broad host range pathogens infecting multiple plant families were more commonly observed on acacias and eucalypts than on the pines (Fig. 3). From these inventories we can derive two important patterns. Firstly, although the non-native pathogens were largely host-specific among the three genera under South African conditions, many infect one or more other plant genera globally. Secondly, the biotic interactions between the ecologically and evolutionary older conifers and their associated fungi appear to be conservative over time (Fig. 3). This is because the non-native pathogens that caught up with pines in South African plantations are globally considered as conifer-specific [see **Supporting Information—Table S1**]. This increases the relevance of risk-assessment based on phylogeny in the recipient community (Fig. 2).

### Insect pest accumulation

The Enemy Release Hypothesis postulates the link between the invasion success of plants and their release from natural enemies found in their native ranges (Keane and Crawley 2002). Large-scale acacia plantations in South Africa appear to have accumulated no accidentally introduced co-evolved insect pests from Australia. *Acacia* plantations also had the lowest number of non-native insect pests among the three genera studied. This relatively higher level of enemy-free space might help to explain their invasive success. In comparison, invasive acacia populations outside of plantations show evidence of some enemy catch-up from Australia, most noticeably the psyllids *Acizzia uncatoides* and *A. acaciaebaileyanae* that both feed on a variety of genera in the Fabaceae (Impson *et al.*, 2009; Percy *et al.*, 2012; Martoni *et al.*, 2016). Furthermore, the Australian scale insect *Icerya purchasi* which, besides feeding on acacias, is also a serious pest of citrus, was found on small populations of *A. paradoxa* in the Western Province of South Africa (Zenni *et al.*, 2009). However, these insects do not appear to significantly impact invasive acacia populations, nor have they yet been recorded as a pest of forestry plantations of acacias. Thus, while many acacias have been introduced for plantation forestry purposes, none of these have experienced insect catch-ups. On the other hand, those enemies that did catch-up with non-forestry acacias appear to have had relatively low impacts in reducing their invasiveness.

Native South African pest accumulation was the highest on acacia plantations. However, among the three studied plantation genera, acacias are the least damaged by insect pests (Roux *et al.*, 2012). Indeed, pathogens of widely planted *A. mearnsii* trees are markedly more harmful than its insect pests (Wingfield *et al.*, 2011). The future role of native herbivore pressure in reducing acacia invasions outside of plantations, therefore, appears weak (Levine *et al.*, 2004; Bezemer *et al.*, 2014; Sunny *et al.*, 2015).

Acacias and eucalypts shared fewer native insect pests than either of them shared with pines (Table 3). This suggests pines are targeted more often by opportunistic native insects. Non-native plants, phylogenetically distinct from native flora, are likely to be utilized by more generalist or opportunistic arthropods (Tallamy 2004; Burghardt and Tallamy 2015). The observation from South Africa with the introduction of *Pinus* into a recipient community with markedly low conifer diversity supports these findings. This poses an intriguing hypothesis; whereby acacias and eucalypts may have similarly evolved plant defence strategies as phylogenetically related native taxa in the recipient region (Burghardt and Tallamy 2015).

Based on the studied insect pest records, the likelihood of a native or non-native insect to feed on acacias, eucalypts and pines appeared to be unrelated to the phylogenetic relatedness to the native flora (Fig. 2). Rather, polyphagous feeding behaviour by native insects appears to have driven this pattern. Conversely, being host-specific (at genus level at least) drove this observation in non-native pests. The similarity in native insect pest communities feeding on these three genera suggests host associations by these generalist pests might occur randomly in space and time. In fact, native insects generally do not impact on these forestry trees compared to the more specialized non-native insects catching up with their lost hosts (Roux *et al.*, 2012). Thus, these non-native trees might present merely an abundant resource to opportunistic native polyphagous insects (Jaenike 1990; Tallamy 2004; Bezemer *et al.*, 2014).

Opportunistic feeding events by some native insects might be especially true for pests of crop establishment such as scarab larvae and locusts (Roux *et al.*, 2012; Harrison and Wingfield 2015). These establishment pests typically feed on roots of saplings, and also ring-bark the soft tissue of the young plants as soon as they are planted into the landscape (Roux *et al.*, 2012). For these insects to become significant pests, saplings must occur in soils where the insects occur in high density. Otherwise they are unlikely to become significant pests at later stages of tree growth, as they do not feed on adult plants.

Vast tree plantations supply ample resources for introduced, host-specific pest species, and given the suitable environmental conditions, they could lead to explosive insect populations (Wright 1983). Such specialist non-native insects are also potentially released from resource competition or parasites in the novel environment (enemy release hypothesis). It is, therefore, unsurprising that when non-native (and co-evolved) catch-ups do occur, the effects on the planted trees can be devastating (Wingfield *et al.*, 2015).

## Patterns of Enemy Accumulation and Invasion Success

**Figure 4.**
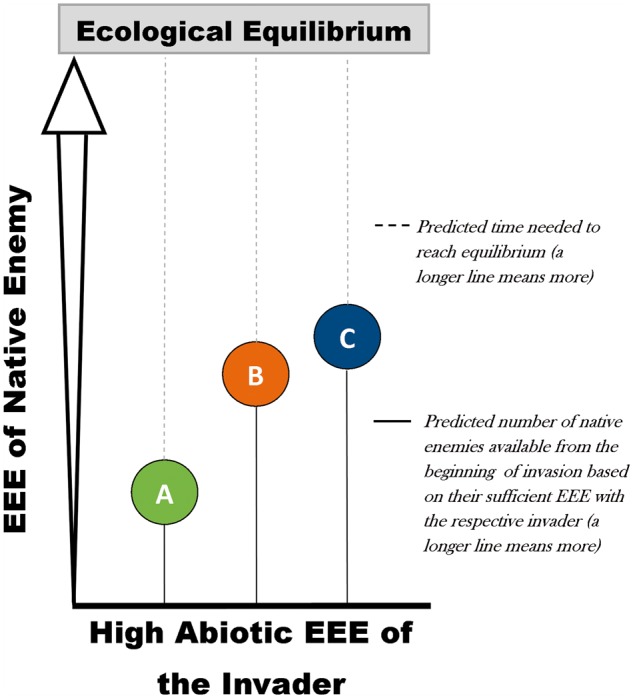
Relative time delay in pathogen accumulation on non-native tree species that have high abiotic eco-evolutionary experience (EEE; see main text) with the novel abiotic environment (e.g. plantation trees pre-selected to flourish in their introduced environments), but the EEE of the recipient native enemy community to utilize the tree as a resource varies. For example, in a model of reduced abiotic limitations, species A, B and C all operate from a high level of EEE within the abiotic environment due to pre-selection but, importantly, may still vary in phylogenetic relatedness to one another and to flora in the novel environment. Consequently, native enemies have different levels of EEE with species A, B and C, resulting in different rates of enemy accumulation and durations until ecological equilibrium is reached. The relativity of enemy accumulation is thus an important phenomenon that would help to predict which species are more vulnerable to disease in the short term. For the South African example studied here, species A may represent pines, species B, acacias, and species C is eucalypts. The role of non-native catch-up may substantially reduce this time delay in reaching equilibrium. This highlights the importance of biosecurity measures to slow biological invasions and to protect crops.

If non-native plants were pre-selected to flourish under prevailing abiotic conditions in the introduced environment, i.e. having high abiotic EEE, then variation in ecological disequilibrium conditions can be explained by the sufficient EEE of native enemies with the respective invader. The low EEE of native pathogens with pines, for example, would further lead us to expect variable accumulation patterns of pathogens onto non-native gymnosperms and angiosperms in South Africa. Ecological disequilibrium can, thus, also be articulated from a relative time delay perspective (Fig. 4). Of course, ecological disequilibrium conditions among non-native trees should theoretically reach equilibrium over evolutionary timeframes as multiple biotic interactions are re-established through reciprocal adaptation (Holt 2009; Zenni *et al.*, 2016, this issue). For example, phylogenetically related non-native and native tree genera had similar numbers and types of ecological interactions with pathogens, and this only after only a few centuries (Vacher *et al.*, 2010).

The finding that a relative time-delay exists in enemy accumulation among diverse non-native flora may help to predict invasion outcomes under current and future conditions (Flory and Clay 2013; Flory and D’Antonio 2015). However, although ecological disequilibrium conditions can contribute to such predictions, on their own they do not necessarily dictate the level and speed by which invasive trees can transform landscapes (homogenize native diversity). For example, acacia invasions in South Africa are generally considered the most aggressive in facilitating landscape homogenization (Le Maitre *et al.*, 2011; Hui *et al.*, 2014). Yet, pines, which are successful invaders in their own right, appear to have benefitted more from native pathogen release. This variation in speed of landscape transformation suggests factors other than enemy release, e.g. species-specific life-history strategies, also contribute to invasion success and population proliferation (Keane and Crawley 2002; Colautti *et al.*, 2004; Lorenzo *et al.*, 2010).

Introduced acacias in South Africa produce massive banks of soil-stored seeds conferring persistence in disturbed environments and propagule pressure to facilitate spread (Richardson and Cowling 1992; Richardson and Kluge 2008). They also have functional traits giving them an advantage over native species in invaded ecosystems. For example, *A. mearnsii* has the ability to adapt both anatomically and physiologically *in situ* to different moisture-availability regimes (Crous *et al.*, 2012*a*, *b*). Acacias are also fast-growing and able to exploit soil nutrients in nutrient-poor environments (Morris *et al.*, 2011), further aided by their ability to fix atmospheric nitrogen through symbiosis with rhizobia (Le Roux *et al.*, 2016, this issue). Furthermore, invasive *Acacia* species in South Africa have very wide native ranges in Australia. This could underpin their ecophysiological and life-history advantages in the many ecosystems to which they have been introduced around the world (Richardson *et al.*, 2011; Hui *et al.*, 2011, 2014). This fact may have helped them to create a potentially large niche hypervolume for establishment in South Africa and elsewhere (Hui *et al.*, 2014). This underscores the complementary nature of integrating hypotheses such as EEE, ecological disequilibria, temporal scales, and *in situ* plant functional or life-history traits to disentangle the variation in tree invasions across multiple genera.

## Implications for Biological Control of Non-Native Trees

### Control by native insect pests

There are limited reports of native insects aiding in the control and spread of invasive trees in South Africa. The native hemipteran *Zulubius acaciaphagus* was found to feed on the seeds of the highly invasive *Acacia cyclops* (Holmes and Rebelo 1988). Similarly, the native and polyphagous moth *Imbrasia cytherea* became a pest on introduced *Pinus* species in South Africa (reviewed in Roux *et al.*, 2012). However, although native herbivore pests may provide some biological control of invasive plants, especially for some acacias (Kaplan *et al.*, 2012), significant damage by native herbivores in general is lacking (Procheş *et al.*, 2008). Native pests, therefore, do not appear to be effective in curbing the spread of acacias, eucalypts and pines in South African ecosystems. Indeed, although native generalist insects are theoretically able to help impact on some non-native tree populations (Sunny *et al.*, 2015), it is unlikely they can actually significantly impact exotic tree abundance as a natural mitigating factor (Maron and Vilà 2001; Levine *et al.*, 2004; Parker *et al.*, 2006; Bezemer *et al.*, 2014).

The inability of generalist native insects to impede invasion is especially true in environments where invasive plant populations are already established in high abundance and over large areas (Maron and Vilà 2001). Being initially released from specialized pests and pathogens (Wingfield *et al.*, 2011), is a precondition of the ‘evolution of increased competitive ability’ or ‘grow vs. defend’ hypotheses (Blossey and Nötzold 1995; Callaway and Ridenour 2004). During the enemy-free phase of invasions, plants would have the ability to more rapidly increase their population size as they spend fewer resources on defence. As a result, an invasive plant population may reach a density threshold where the suppressing effects of native polyphagous pests become negligible (Maron and Vilà 2001).

### Control by native pathogens

The use of native pathogens in biological control of invasive trees is an interesting prospect. For example, the native fungus *Pseudolagarobasidium acaciicola* has been proposed as a potential mycoherbicide for the invasive tree *Acacia cyclops* in South Africa (Wood and Ginns 2006; Kotzé *et al.*, 2015). Similarly, the native pathogen *Colletotrichum acutatum* was suggested and is used as a mycoherbicide for the non-native and invading shrub *Hakea sericea* (Gordon and Fourie 2011). However, because native host-shifts onto non-native trees appear to be rare globally (Mitchell and Power 2003), mycoherbicide development is likely to be a slow process. Native pathogens in South Africa might, therefore, provide some control of invasive populations of acacias and eucalypts. But similar to native insect pests, might not be a viable management option due to the relative time delay in fungi manifesting as pathogens. However, artificially selecting native pathogens to more rapidly develop mycoherbicides might mitigate such time delays.

### Control by non-native insect pests and pathogens

Specialized or co-evolved non-native insect pests and pathogens would damage invasive acacia, eucalypt and pine populations outside of plantations should they spread beyond plantation boundaries. Evidence for using non-native host-specificity as a proxy for biological control is plentiful in the biological control literature. Of the 106 non-native biological control agents (including insects and pathogens) introduced to curb the spread of invasive non-native plants in South Africa, 75 have established and most of these have suppressed the focal invasive species (Klein 2011; Moran *et al.*, 2005, 2013). The control of the invasive *Acacia saligna* using a co-evolved fungus, *Uromycladium tepperianum*, provides an example of exploiting fungal host-specificity to decrease population numbers (Wood and Morris 2007). Indeed, for invasive Australian acacias in particular, there has been considerable progress in introducing only co-evolved and host-specific insect pests to reduce non-target effects (Impson *et al.*, 2011). In turn, the tight host-specificity and population decimation observed in the unintentionally introduced non-native insect pests onto eucalypts and pines in particular (Wingfield *et al.*, 2008; Roux *et al.*, 2012), provides a theoretical basis as to why biological control initiatives using these insect pest catch-ups can help to mitigate tree invasions in the plantation forestry matrix.

**Why have insect pest catch-ups not been impacting invasive populations in the plantation matrix?:** Conceivably, non-native insect pest catch-ups have spread beyond the plantations. Yet, evidence of these catch-ups impacting on naturalized populations of acacias, eucalypts or pines, remains to be quantified. A possible explanation as to why catch-ups outside of plantations are so low is that these forest plantation pests are usually under biological control (Garnas *et al.*, 2016). Plantations in which the pests are under biological control can, thus, be seen as maintaining the necessary carrying capacity (resources) to prevent these catch-ups fully establishing in plantation matrices (species-energy theory; Wright 1983). In this case, spill-over of non-native insect pest accumulation as a source of controlling plant invasion in plantation matrices might be unreliable.

### Integrating biotic and abiotic approaches to control non-native flora

The physical removal of invasive trees and the active rehabilitation of natural biotic communities have been suggested as a more effective invasion management strategy than relying on enemy accumulation over time (Parker *et al.*, 2006). For example, in the hyperdiverse fynbos biome of South Africa, the removal of invasive *A. mearnsii* trees from riparian zones, and the subsequent recovery of native plant diversity, helped to recover both alpha and beta arthropod diversity (Maoela *et al.*, 2016). However, as recently shown, pathogen accumulation on a highly invasive grass in the US may also help to significantly reduce the fitness of invasive populations in the future (Stricker *et al.*, 2016). Thus, a combination of using enemies (particularly native pathogens that have undergone host-shifts and agents selected for biological control) and reducing the invasive debt through physical eradication of the trees should be highly complementary approaches to curb the spread of aggressive invaders in South Africa (Wilson *et al.*, 2011).

## Contrasting Implications of Ecological Disequilibria for Forestry and Biodiversity Management

In South Africa, invasive acacias, eucalypts and pines have substantial negative impacts on biodiversity (Richardson and Van Wilgen 2004; Le Maitre *et al.*, 2011), stream flow from water catchments (Bosch and Hewlett 1982; Le Maitre *et al.*, 1996; Dye *et al.*, 2001; Dye and Jarmain 2004), and water quality (Chamier *et al.*, 2012; Tye and Drake 2012). This represents a dilemma as plantation forestry is a part of many South African agricultural landscapes with significant socio-economic benefits, critically important for a developing nation (Van Wilgen and Richardson 2012). Ecological disequilibria caused by pathogens and insect pests might, thus, have contrasting implications for managing invasive species spread or crop health.

### Pathogens

If we accept that control of invasive species by natural enemies is an important ecosystem service (Mitchell and Power 2003), then native pathogens appear to fall outside the scope of an ecosystem service provider to naturally and rapidly mitigate invasion of acacias, eucalypts and pines. Conversely, for commodity production, e.g. using conifers such as *Pinus* in a landscape where gymnosperms are depauperate, this absence of native pathogens limits product losses. Non-native pathogen catch-up events may reduce invading populations outside of plantations (Mitchell *et al.*, 2006; Flory and Clay 2013; Stricker *et al.*, 2016). But this could be risky to local biodiversity and local ecosystem function given the observed phylogenetic link (relatedness between native and non-native species) to host shifts (Fig. 2; see also Bufford *et al.*, 2016). The probability of spill-over effects of non-native pathogens into native ecosystems remains topical and in need of critical examination (Flory and Clay 2013; Blackburn and Ewen 2016; Bufford *et al.*, 2016; Stricker *et al.*, 2016).

There are thus contrasting implications of ecological disequilibrium conditions to invasion management and plantation forestry. Nonetheless, from both a forestry and biological invasions perspective, there is a need to more effectively control the establishment of novel non-native pathogens, particularly those pathogens associated with trees more phylogenetically related to the South African flora, such as acacias and eucalypts. This is because pathogens of acacias and eucalypts are presently destroying wood products and have the potential to threaten native flora via host-shifting events in the future (Burgess and Wingfield 2017).

An important example of the future threat from evolutionary closely related species to both plantation forestry and native ecosystems is found in species from the Myrtaceae. The myrtle rust fungus, *Puccinia psidii*, is native on Myrtaceae in South and Central America (Coutinho *et al.*, 1998; Glen *et al.*, 2007). This rust has shown preference for non-native Myrtaceae including *Eucalyptus*, and is considered to be a major threat to native eucalypt ecosystems and plantations globally (Glen *et al.*, 2007). *Puccinia psidii* has recently also been found on native forest Myrtaceae in South Africa (Roux *et al.*, 2013; 2015). Given high numbers of native Myrtaceae species in South Africa, this invasion pattern suggests many native trees might be ‘collateral damage’ of the increase in pathogen catch-up events onto eucalypts.

### Insect pests

Fortunately, there is no evidence that any of the non-native insect pests having accidentally caught up with their hosts in South African plantations have started to utilize native flora. This most likely reflects the host-specificity of these non-native insect taxa (Fig. 2), and is akin to the host-specificity and thus biological control efficacy of deliberately introduced insect pests (e.g. Impson *et al.*, 2011; Hajek *et al.*, 2016). Nonetheless, pests and pathogens accidentally introduced into planted landscapes could over time present a worrying scenario where both natural ecosystem goods (Boyd *et al.*, 2013) and planted ecosystem goods are negatively affected (Wingfield *et al.*, 2015). As a result, managing the impacts of accumulating biological invasions for both ecosystem and commodity conservation would require an on-going collaboration between conservation agencies and production companies (Van Wilgen and Richardson 2014).

## Ecological Disequilibrium Conditions in the Era of Global Connectivity

Globalization and a free-market economy have led to increased transfer in organisms between countries (Jenkins 1996; Westphal *et al.*, 2008; Banks *et al.*, 2015). For example, the Chinese economic ‘boom’ over the last two decades has been accompanied by a rapid increase in the transfer of biota with trading partners (Ding *et al.*, 2008). The invasion of non-native pests and pathogens has also increased dramatically in the last few decades (Liebhold *et al.*, 2012, Santini *et al.*, 2013).

In South Africa, a diversity of plantation genera begets a diversity of non-native pest/pathogen catch-ups. Furthermore, non-native pathogens, which depend on many external factors such as accidental introduction, are more likely to accumulate on the non-native trees than are native pathogens through time. As it stands, invasions of insect pests and pathogens would significantly increase in time (Taole *et al.*, 2015; Garnas *et al.*, 2016), and will be sustained by the presence of healthy and expanding populations of non-native plant hosts. More worryingly, commodity production landscapes could also expect multiple accidental introductions from a single insect pest or pathogen species which might increase genetic diversity and resilience of the pest or pathogen (Taole *et al.*, 2015; Garnas *et al.*, 2016).

Non-native pathogens with a broader host-range would need less random events of dispersing to the right host at the right time (Parker and Gilbert 2004). Known pathogen genera infecting a wide variety of plant families should therefore be especially prioritized when managing import-export protocols. Particular functional groups of pathogens such as canker and wilt pathogens are historically more likely to become invasive (Burgess *et al.*, 2016, this issue). Highly specialized insect pests are also successful in locating and negatively affecting their lost hosts. This is especially disconcerting since there is a vast community of potential insects already known to damage similar plantation forestry species in other countries (Paine *et al.*, 2011; Hurley *et al.*, 2016). Maintaining the *status quo* in trading regulation, these pests are likely to also spread to South Africa.

Native polyphagous insect pests feeding on non-native trees could be seen as gaining experience with a new food resource, especially when exploring it more frequently due to the abundance of the resource. Insect species are able to adapt to utilize novel hosts via transgenerational acclimatization (Cahenzli *et al.*, 2015), which is seen as a positive response to exploit readily available resources in the landscape, and ultimately increase the fitness of a species (Jaenike 1983, 1990; Cahenzli *et al.*, 2015). Should these species spread to the country of origin of the invasive tree, and should the environmental conditions also be conducive to establishment, these formerly polyphagous insects could even become invasive and enemies of that particular tree species in its native range.

The potential for reciprocal exchange in pests and pathogens between countries provides a further argument as to why trading in commodities should be strictly controlled at both the import and export level. For example, in South Africa, the native and polyphagous scarab beetle, *Heteronychus arator* (black maize beetle), was occasionally recorded as a minor pest of eucalypts (Govender 2005). In turn, *H. arator* is considered one of the most damaging pests of *Eucalyptus globulus* plantations in Australia (Loch and Floyd 2001). This reciprocity perhaps reflects the breadth of how difficult biological invasions are to manage at the global scale. Still, connectivity between environmentally similar countries or regions may be even more prone to such events. Since eucalypts and pines are globally widely planted commodities, such a continuous host population will most likely act as sources for novel pests and pathogens. The numbers of catch-up and host-shift events between three major planted tree genera provides further compelling evidence to limit or at least better regulate the import and export activities to reduce commodity losses and biodiversity decline (Wingfield *et al.*, 2011, 2015; Liebhold *et al.*, 2012; Santini *et al.*, 2013; Hurley *et al.*, 2016).

## Conclusions

The accumulation patterns of native and non-native pests and pathogens onto *Acacia*, *Eucalyptus* and *Pinus* plantations in South Africa varied considerably. Non-native trees in these genera might thus be under various conditions of ecological disequilibrium, which could enhance their potential establishment and spread in the introduced environments. Importantly, native enemy release may be transient and have a distinct lag phase (Facon *et al.*, 2006), but depending on the phylogenetic relatedness of the host lineage (invader or commercial species) to the native flora, some enemy-free phases might last longer than others. For example, there appears to be very little chance of relatively host-specific or specialized native fungal pathogens attacking pines in South Africa, which have no confamilial relatives in the region*.* To the contrary, acacias and eucalypts, which have many confamilial relatives in the region, already accumulated more native pathogens. This pattern was, however, different for native polyphagous or opportunistic insect pests, which have accumulated on all three host genera.

Due to possible convergent evolution in plant traits (Ackerly and Reich 1999), enemy accumulation might not always be related to the phylogenetic relationship between the donor and the local flora. In this light, using the EEE concept would allow for the integration of key abiotic and biotic interactions and adaptations that should influence enemy accumulation, e.g. phylogenetic relatedness, convergent evolution and habitat similarity. This concept thus provides a valuable framework to explain and predict ecological disequilibria.

There is a pressing need for more rapid responses to manage novel plant invasions (Simberloff *et al.*, 2013). Yet, in an era of global connectivity, it can be difficult to predict when and where invasion events will occur. This is an important underlying reason why we still underestimate the accumulating effects that biological invasions might have on ecosystem function and crop health; an oversight that may be very expensive to mitigate later (Essl *et al.*, 2015). Retrospectively analysing pest and pathogen accumulation on established non-native flora (Flory and D’Antonio 2015), in order to populate EEE frameworks, can help to 1) assess which native plant genera are likely to accumulate introduced enemies in the shortest time; and 2) determine the likelihood of commercially-important tree species experiencing disease-related productivity loss from native pathogens. This latter fact also emphasises the importance of biosecurity measures to reduce the chances of accidentally introducing insect pests and pathogens of non-native crop plants.

## Sources of Funding

This paper had its origin at a workshop on ‘Evolutionary dynamics of tree invasions’ hosted by the DST-NRF Centre of Excellence for Invasion Biology (C•I•B) in Stellenbosch, South Africa, in November 2015. Funding for the workshop was provided by the C•I•B, Stellenbosch University (through the office of the Vice Rector: Research, Innovation and Postgraduate Studies), and the South African National Research Foundation (DVGR grant no. 98182). CJC, BS and MJW also thank the members of the Tree Protection Co-operative Programme (TPCP) and the DST-NRF Centre of Excellence in Tree Health Biotechnology at FABI, University of Pretoria, for additional funding. Murdoch University through the Sir Walter Murdoch Scheme (awarded to MJW) supported the travel of TIB to attend the workshop.

## Contributions by the Authors

All authors contributed significantly to the conceptualization, discussion and subsequent writing of this review.

## Conflicts of Interest Statement

None declared.

## Supplementary Material

Supplementary Data

## Supporting Information

The following additional information is available in the online version of the article —

**Table S1.** List of analyzed insect pests and pathogens associated with damage in *Acacia*, *Eucalyptus* and *Pinus* plantations in South Africa (Wingfield *et al.*, 2008; Roux *et al.*, 2012).
