# Supplementary material for: Ecological disequilibrium drives insect pest and pathogen accumulation in non-native trees
Source: AoB Plants. 2016 Dec 23;9(1):plw081. doi: 10.1093/aobpla/plw081 (PMC5499825; doi:10.1093/aobpla/plw081)
Supplement: Supplementary Data [file plw081_Supp.docx]

**Table S1.** List of analyzed insect pests and pathogens associated with damage in *Acacia*, *Eucalyptus* and *Pinus* plantations in South Africa (Wingfield *et al*. 2008; Roux *et al*. 2012).

| **NON-NATIVE PATHOGENS** |  |  |  |  |  |
| --- | --- | --- | --- | --- | --- |
|  | ***Acacia*** | ***Eucalyptus*** | ***Pinus*** | ***Polyphagous/Host-specific**** | ***Angiosperm or Gymnosperm specific?*** |
| *Aulographina eucalypti* | 0 | 1 | 0 | Host-specific | Angiosperm-specific |
| *Botryosphaeria dothidea* | 1 | 1 | 0 | Polyphagous | Both |
| *Camptomerris albizziae* | 1 | 0 | 0 | Host-specific | Angiosperm-specific |
| *Cercoseptoria pini-densiflorae* | 0 | 0 | 1 | Host-specific | Gymnosperm-specific |
| *Cyclaneusma minus* | 0 | 0 | 1 | Host-specific | Gymnosperm-specific |
| *Cylindrocladium pauciramosum* | 0 | 1 | 0 | Polyphagous | Angiosperm-specific |
| *Cytospora aff. austromontana* | 0 | 1 | 0 | Host-specific | Angiosperm-specific |
| *Cytospora chrysosperma* | 0 | 1 | 0 | Host-specific | Angiosperm-specific |
| *Cytospora eucalypticola* | 0 | 1 | 0 | Host-specific | Angiosperm-specific |
| *Diplodia pinea* | 0 | 0 | 1 | Host-specific | Gymnosperm-specific |
| *Dothistroma septosporum* | 0 | 0 | 1 | Host-specific | Gymnosperm-specific |
| *Erythricium salmonicolor* | 1 | 1 | 0 | Polyphagous | Angiosperm-specific |
| *Fairmaniella leprosa* | 0 | 1 | 0 | Host-specific | Angiosperm-specific |
| *Fusarium circinatum* | 0 | 0 | 1 | Host-specific | Gymnosperm-specific |
| *Holocryphia eucalypti* | 0 | 1 | 0 | Host-specific | Angiosperm-specific |
| *Kirramyces epicoccoides* | 0 | 1 | 0 | Host-specific | Angiosperm-specific |
| *Kirramyces eucalypti* | 0 | 1 | 0 | Host-specific | Angiosperm-specific |
| *Lophodermium spp.* | 0 | 0 | 1 | Host-specific | Gymnosperm-specific |
| *Neofusicoccum spp.* | 1 | 1 | 0 | Polyphagous | Angiosperm-specific |
| *Pantoea ananatis* | 0 | 1 | 0 | Polyphagous | Angiosperm-specific |
| *Phytophthora cinnamomi* | 1 | 1 | 0 | Polyphagous | Both |
| *Pythium splendens* | 0 | 1 | 0 | Polyphagous | Both |
| *Quambalaria eucalypti* | 0 | 1 | 0 | Host-specific | Angiosperm-specific |
| *Ralstonia solanacearum* | 0 | 1 | 0 | Polyphagous | Angiosperm-specific |
| *Rhizina undulata* | 0 | 0 | 1 | Polyphagous | Gymnosperm-specific |
| *Sphaerotheca pannosa* | 1 | 1 | 0 | Host-specific | Angiosperm-specific |
| *Teratosphaeria nubilosa* | 0 | 1 | 0 | Host-specific | Angiosperm-specific |
| *Teratosphaeria zuluense* | 0 | 1 | 0 | Host-specific | Angiosperm-specific |
| *Uromycladium acaciae* | 1 | 0 | 0 | Host-specific | Angiosperm-specific |
| *Valsa aff. cinereostroma* | 0 | 1 | 0 | Polyphagous | Angiosperm-specific |
| *Valsa fabianae* | 0 | 1 | 0 | Host-specific | Angiosperm-specific |
| *Xanthomonas spp.* | 0 | 1 | 0 | Polyphagous | Angiosperm-specific |
|  |  |  |  |  |  |
|  |  |  |  |  |  |
| **NATIVE PATHOGENS** |  |  |  |  |  |
|  | ***Acacia*** | ***Eucalyptus*** | ***Pinus*** | ***Polyphagous/Host specific**** | ***Angiosperm or Gymnosperm specific?*** |
| *Armillaria fuscipes* | 1 | 1 | 1 | Polyphagous | Both |
| *Ceratocystis albifundus* | 1 | 0 | 0 | Polyphagous | Angiosperm-specific |
| *Crysoporthe austroafricana* | 0 | 1 | 0 | Host-specific | Angiosperm-specific |
| *Pseudophaeolus baudonii* | 0 | 1 | 0 | Polyphagous | Angiosperm-specific |

*Polyphagous – Infecting multiple plant families, whereas being host-specific was classified as infecting within a plant family.

| **NON-NATIVE INSECT PESTS** |  |  |  |  |
| --- | --- | --- | --- | --- |
|  | ***Acacia*** | ***Eucalyptus*** | ***Pinus*** | ***Specialist/Generalist*** |
| *Apate indistincta* | 1 | 0 | 0 | Generalist |
| *Blastopsylla occidentalis* | 0 | 1 | 0 | Specialist |
| *Cinara cronartii* | 0 | 0 | 1 | Specialist |
| *Ctenarytaina eucalypti* | 0 | 1 | 0 | Specialist |
| *Eulachnus rileyi* | 0 | 0 | 1 | Specialist |
| *Glycaspis brimblecombei* | 0 | 1 | 0 | Specialist |
| *Gonipterus scuttelatus* | 0 | 1 | 0 | Specialist |
| *Hylastes angustatus* | 0 | 0 | 1 | Specialist |
| *Hylurgus ligniperda* | 0 | 0 | 1 | Specialist |
| *Leptocybe invasa* | 0 | 1 | 0 | Specialist |
| *Orthotomicus erosus* | 0 | 0 | 1 | Specialist |
| *Phoracantha recurva* | 0 | 1 | 0 | Specialist |
| *Phoracantha semipunctata* | 0 | 1 | 0 | Specialist |
| *Pineus boerneri* | 0 | 0 | 1 | Specialist |
| *Pissodes nemorensis* | 0 | 0 | 1 | Specialist |
| *Sinoxylon bellicosum* | 1 | 0 | 0 | Unknown |
| *Sirex noctilio* | 0 | 0 | 1 | Specialist |
| *Thaumastocoris peregrinus* | 0 | 1 | 0 | Specialist |
| *Trachymela tincticollis* | 0 | 1 | 0 | Specialist |
| *Xyleborinus spp.* | 0 | 0 | 1 | Unknown |
| *Xyleborus spp.* | 0 | 0 | 1 | Unknown |
|  |  |  |  |  |
| **NATIVE INSECT PESTS** |  |  |  |  |
|  | ***Acacia*** | ***Eucalyptus*** | ***Pinus*** | ***Specialist/Generalist*** |
| *Achaea lienardi* | 1 | 0 | 0 | Generalist |
| *Agrotis spp.* | 1 | 1 | 1 | Generalist |
| *Catamonus spp.* | 1 | 0 | 0 | Unknown |
| *Chaliopsis junodi* | 1 | 0 | 0 | Generalist* |
| *Cleora herbuloti* | 0 | 1 | 1 | Unknown |
| *Colasposoma spp.* | 0 | 1 | 1 | Unknown |
| *Coryphodema tristis* | 0 | 1 | 0 | Generalist |
| *Ellimenistes laesicollis* | 0 | 1 | 0 | Unknown |
| *Euproctis terminalis* | 0 | 0 | 1 | Generalist* |
| *Gynanisa maia* | 1 | 0 | 0 | Generalist |
| *Hypopholis sommerii* | 1 | 0 | 1 | Generalist |
| *Imbrasia cytherea* | 1 | 0 | 1 | Generalist* |
| *Lygidolon laevigatum* | 1 | 0 | 0 | Unknown |
| *Monochelus calcaratus* | 1 | 0 | 0 | Unknown |
| *Pachypasa capensis* | 1 | 1 | 1 | Generalist* |
| *Pseudobunea irius* | 1 | 1 | 1 | Unknown |
| *Various grasshoppers and crickets*** | 1 | 1 | 1 | (morphogroup) |
| *Various Scarabaeidae larvae*** | 1 | 1 | 1 | (morphogroup) |
| *Various Hodotermitidae*** | 1 | 1 | 0 | (morphogroup) |

*Generalist, and known to feed on both angiosperms and gymnosperms

**Morphogroups were treated as 'species' in the accumulation patterns for insects, since there lacks data on the exact species involved. Many minor or rarely observed insect pests may have been omitted here; our focus is on commonly known plantation forestry pests during the past century.

***These inventories (pathogens and insect pests) were mainly from the following species (and hybrids) within each genus: *Acacia*: *A. mearnsii*, *A. decurrens*; *Eucalyptus*: *E. camaldulensis, E. grandis, E. urophylla, E. nitens, E. smithii, E. dunni, E. saligna; Pinus: P. radiata, P. taeda, P. ellioti, P. patula*
